# Epidemiological evidence of higher susceptibility to vCJD in the young

**DOI:** 10.1186/1471-2334-4-26

**Published:** 2004-08-10

**Authors:** Pierre-Yves Boëlle, Jean-Yves Cesbron, Alain-Jacques Valleron

**Affiliations:** 1 INSERM U444, Assistance Publique Hôpitaux de Paris, Université Pierre et Marie Curie, Paris, France; 2 Immunité Anti-Infectieuse JE 2236, UFR de Médecine de Grenoble, Université Joseph Fourier, Grenoble, France

## Abstract

**Background:**

The strikingly young age of new variant Creutzfeldt-Jacob disease (vCJD) cases remains unexplained. Age dependent susceptibility to infection has been put forward, but differential dietary exposure to contaminated food products in the UK population according to age and sex during the bovine spongiform encephalopathy (BSE) epidemic may provide a simpler explanation.

**Methods:**

Using recently published estimates of dietary exposure in mathematical models of the epidemiology of the new variant Creutzfeldt Jacob disease (vCJD), we examine whether the age characteristics of vCJD cases may be reproduced.

**Results:**

The susceptibility/exposure risk function has likely peaked in adolescents and was followed by a sharp decrease with age, evocative of the profile of exposure to bovine material consumption according to age. However, assuming that the risk of contamination was proportional to exposure, with no age dependent susceptibility, the model failed to reproduce the observed age characteristics of the vCJD cases: The predicted cumulated proportion of cases over 40 years was 48%, in strong disagreement with the observed 10%. Incorporating age dependent susceptibility led to a cumulated proportion of cases over 40 years old of 12%.

**Conclusions:**

This analysis provides evidence that differential dietary exposure alone fails to explain the pattern of age in vCJD cases. Decreasing age related susceptibility is required to reproduce the characteristics of the age distribution of vCJD cases.

## Background

Owing to the lack of data concerning age dependent dietary exposure to bovine material in food in the UK, mathematical models used in the study of new variant Creutzfeldt-Jacob disease (vCJD) have postulated an age risk function for contamination where the influence of age dependent dietary exposure to contaminated material could not be separated from intrinsic age related susceptibility [1-3]. For example, we modelled exposure/susceptibility by a plateau during the first 15 years of age followed by an exponential decrease afterwards [2]. Using this approach we estimated the duration of the incubation period for vCJD to circa 15 years; and predicted that the epidemic had likely peaked in 2001–2002 which is now consistent with the observed data [2,3]. The simple representation of the age risk function implied that few parameters were necessary to describe it, a desirable property when estimation is based on limited data.

By June 2003, 139 cases of vCJD had been reported to the vCJD surveillance unit in the UK, as compared to the 79 cases used in our original paper [2]. This warrants the inclusion of more detail in the age risk function, especially to investigate whether the constant risk assumption in children holds, as it led to predict a bimodal age distribution for cases in the coming years [3]. At the same time, detailed estimates of the dietary bovine material consumption in the UK population according to age and sex have been made available [4]. This offers the opportunity to try to disentangle the role of exposure from that of age-specific susceptibility. Indeed, the estimates for dietary exposure show that it peaked during adolescence and decreased with age afterwards, a pattern which is consistent with the finding that most vCJD cases are young and were therefore at most teenagers during the years when the bovine spongiform encephalopathy (BSE) epidemic was at its maximum.

We therefore set out to estimate the age risk function with a versatile description based on step functions, and to investigate whether dietary exposure alone could explain the age distribution of cases.

## Methods

### Data

We obtained the age, sex and date of onset for the 137 vCJD cases reported to the UK vCJD unit as of June 2003, all of which had onset before October 2002. Delays in reporting may reach 18 months (R Will, personal communication) and bias downward the incidence curve. Therefore, we included in our analysis only the 129 cases with onset before November 2001, consisting in 71 men and 58 women. Mortality by age and sex for the UK was obtained from the Office for National Statistics [5].

### Model

The model extends our previously described method [3]. The instantaneous risk of infection for vCJD, *λ(a,t)*, was assumed to depend on date *t *and age *a *in a multiplicative manner, with *λ(a,t) *= *f(a) g(t)*, where 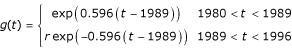 parallels the entry of infected cows in the human food chain estimated from back-calculation results in the UK [6] and *r *corresponds to the impact of the specified risk material ban in 1989; *f(a) *is a function varying with age only (see following paragraph). Occurrence of cases of vCJD of sex *i *(i = Male or Female) is then modelled by a Poisson process in the (age, time) plane, with intensity 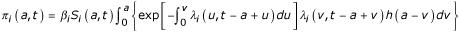, so that the expected number of onsets of sex *i *in the period [*t,t*+d*t*) and age [*a*,*a*+d*a*) is *π*_*i*_(*a*,*t*) d*a *d*t *[2,7]. In the equation above, *h *is the distribution of the incubation time, *S*_*i*_(*a,t*) is the probability of survival at time *t *for individuals of sex *i *aged *a *calculated from census information, and *β*_ι _is the intensity of a homogeneous Poisson process. The incubation period distribution *h *was taken to be lognormal, as the choice of a particular shape is not critical for the estimates [3]. This distribution was also assumed to be independent of sex, and of age at contamination.

Estimates of the parameters were obtained by numerical maximization of the log-likelihood, defined as 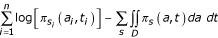, where the first sum is on all observed cases where *s*_*i *_is the sex of individual *i*, and the second is over both sexes (*s*) and requires integration of the intensity over the domain (in time and age) *D *= [0,100] × [1 Jan 1980,31 Nov 2001]. Custom FORTRAN code, using SLATEC and TOMS library for numerical routines, was used in this step. In all instances, our model allowed estimation of the mean and standard deviation of the incubation period, the shape of *f(a)*, and *β*.

The model predicted distribution of age for cases with onset before November 2001 was determined by integrating 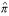
(*a*,*t*) over the period [1/1/1980, 1/11/2001] for consecutive age class of width 10 years. The *χ*^2 ^distance was computed to measure agreement between the observed and the model calculated age distributions, but no formal test was carried out.

### Age Risk function

#### Model "Susceptibility & Diet"

To allow a versatile shape in the age risk function, we chose a non parametric description based on step functions rather than a mathematical function with few parameters.

We wrote 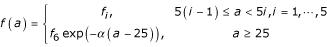 so that the shape of *f(a) *was unconstrained for a<25 and decreased exponentially after age 25. We then estimated *f*_*i*_, *i *= 1,...,6 and *α *at maximum likelihood. With this particular choice, it is possible to visually check the progression in risk with age during infancy (*f*_1_, *f*_2_, *f*_3_), the presence of a peak in risk in teenagers (*f*_2_,*f*_3_,*f*_4_), and whether the drop in risk after 20 is strong enough to be exponential (*f*_4_, *f*_5_, *f*_6_).

#### Model "Diet Alone"

To investigate whether dietary exposure to meat was sufficient to explain the young age of vCJD cases, we first determined the exposure to meat according to age and sex from UK data, using consumption of carcass meat [4]. In these data, it is seen that the quantity consumed by week, as well as the percentage of consumers, changes with age. We therefore defined *e(a) *as the product of mean consumption by the corresponding percentage of consumers in age class *a*, and wrote *f(a) *= *f*_0 _*e(a)*, where *f*_0 _was estimated at maximum likelihood.

#### Model "Susceptibility | Diet"

Finally, to estimate the influence of age standardised on dietary exposure, we wrote 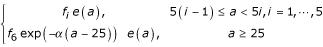 and likewise estimated *f*_*i *_from the data.

In model "**Susceptibility & Diet**" and "**Susceptibility | Diet**", we normalised the *f*_*i *_by the largest of the values in the graphical presentation of results, yielding risks relative to the highest risk age class.

### Confidence intervals

Maximum likelihood estimators have a limiting normal distribution around the true parameters, with limiting variance/covariance matrix given by the inverse of the Fisher information [8], therefore the quadratic form 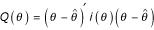, where *θ *stands for a vector of *k *parameters, 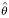 for the estimates, and ***i***(*θ*) for the Fisher information matrix, has a limiting chi-squared distribution with *k *degrees of freedom. We therefore determined 95% confidence intervals by finding the values of parameters so that *Q*(*θ*) was less than the 95^th ^quantile of the corresponding chi-squared distribution.

## Results

**Figure 1:**
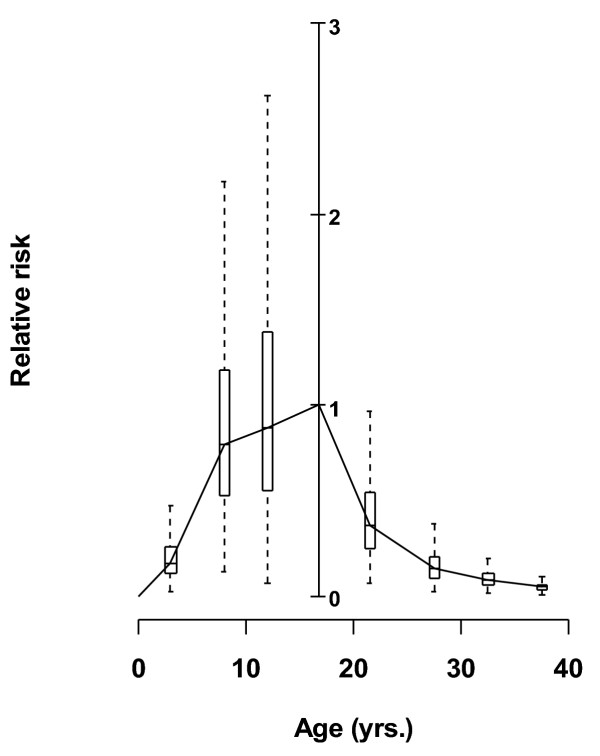
**Age dependent exposure/susceptibility risk function for vCJD infection. **Risks are relative to the [15, 20] years old. Box and whiskers plots show the 50% and 95% confidence intervals.

**Figure 2:**
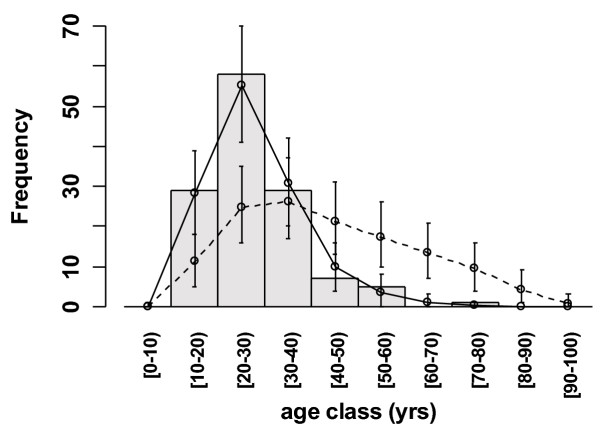
**Observed age distribution of the 129 vCJD cases with onset before November 2001 and model predicted cumulated age distribution of cases for susceptibility proportional to meat consumption only (dashed) or with estimated age risk function (plain). **Bars limit 95% confidence intervals.

The estimated age risk function in model "**Susceptibility & Diet**" is presented in Figure 1, and shows that an increase during childhood, peak during adolescence and sharp decrease afterwards provided the best fit. In this model, the average incubation period was estimated at 13.2 years (CI95% [11.2, 15.8]), with standard deviation 2.0 years (CI95% [1.1,3.7]). The model predicted age for the cases showed good agreement with the observed distribution (*χ*^2 ^= 2.45; Figure 2).

**Figure 3:**
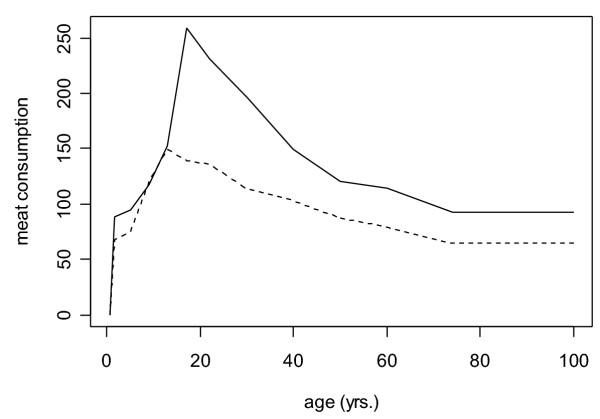
**Average dietary exposure per person per week in the UK according to sex (plain : Male, dashed : Female). **Data from Cooper & Bird [4].

The shape of the estimated age risk function in Figure 1 is evocative of the age profile of dietary exposure to bovine carcass meat in the 1980s in the UK, as shown in Figure 3, where an increase in consumption was noted during childhood and adolescence, and decreased afterwards. However, in model "**Diet Alone**", while the average incubation period was estimated at 12.1 (CI 95% [10.2, 14.2]) years with a standard deviation of 2.4 (CI95% [1.2, 4.1])years, close to that of the first model, the predicted age distribution of the cases was at odds with the observed distribution (*χ*^2 ^= 58.4), as apparent in Figure 2. More precisely, the predicted percentage of cases aged over 40 was 48% with the "**Diet Alone**" assumption, when the observed percentage was only 10%; it was 12% with the "**Susceptibility & Diet**" model.

**Figure 4:**
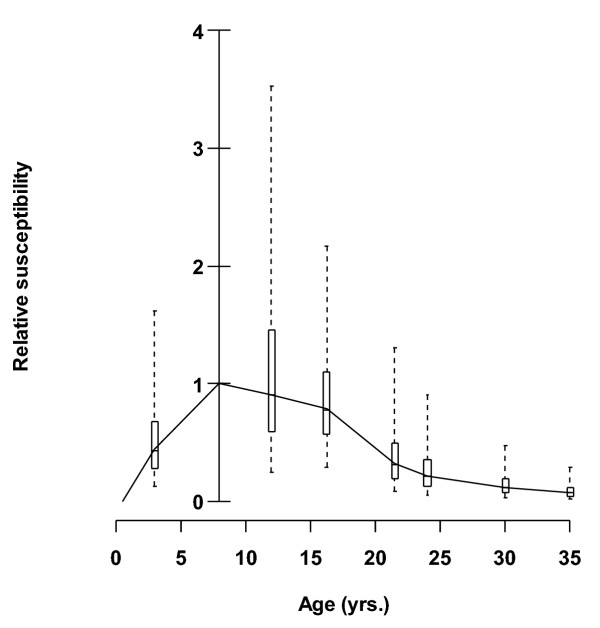
**Age dependent susceptibility risk function for vCJD infection adjusted on dietary exposure to bovine material. **Risks are relative to the [5, 10] years old. Box and whiskers plots show the 50% and 95% confidence intervals.

When estimating the residual influence of age, once exposure has been taken into account, model "**Susceptibility | Diet**" led to a profile for the fi as shown in Figure 4. This profile retained the major characteristics of the age risk function obtained in the "**Susceptibility & Diet**" model, although with an earlier maximum susceptibility. With this model, the average incubation period established at 12.6 years (CI 95% [10.5, 14.7]) with standard error 1.8 years (CI95% [1.2, 3.5]), and the predicted age distribution of cases showed agreement similar to that of the "**Susceptibility & Diet**" model (*χ*^2 ^= 2.36).

## Discussion

Incorporating differential dietary exposure to BSE infected products according to age and sex, in a flexible age risk function for vCJD contamination, we found that exposure alone could not explain the young age of vCJD cases seen in the UK. Decreasing age related susceptibility had to be assumed to reproduce the characteristics of the age distribution of these cases.

In all instances, the estimates for the epidemiological characteristics of vCJD were in line with those previously reported from comparable number of cases [3,9,10], and pointed to an epidemic of moderate size. We obtained a smaller point estimate for the mean incubation period when the age risk was allowed to change during childhood rather than assumed constant (13.2 yrs CI95% [11.2, 15.8] vs. 16.4 yrs CI95% [11, 24] [3]). All models considered here predicted that, by 2010, the epidemic should have ended, provided it is limited to individuals with the observed susceptible genotype (as of today, all vCJD cases are methionine homozygous at codon 129 of the PrP gene[11]). The average number of cases to come is predicted at 43, leading to a total size of 172 cases.

From the model "**Susceptibility & Diet**", it appears that a previous assumption of a constant age risk in children and adolescents [2] has likely led to overestimate the risk of infection in young children. The age-risk profile estimated here leads to a smaller risk than previously found in children born after 1980, to fewer cases among the young and an overall smaller total size for the epidemic than reported before. Furthermore, since few young cases are expected in the future, the bimodality of the predicted age distribution of cases is not found anymore. The estimated profile of the susceptibility/exposure age risk function agreed with that selected in scenario analysis [9], although our maximal risk is among the 15–20 years old rather than in the 10–15 years old.

However, even after adjustment for dietary exposure, susceptibility remains rapidly decreasing in adults, as found in the "**Susceptibility | Diet**" model. This finding confirms that obtained in a recent analysis based on scenario analysis, where it was found that exponential decrease in susceptibility in the oldest cohorts was desirable [10]. This is also found here by direct estimation, and moreover, the age susceptibility appears to increase among children up to age 5–10, be almost constant among teenagers and rapidly decreasing afterwards.

Three hypotheses have been put forward to explain the young age of the vCJD cases: age dependent incubation period, age dependent exposure, and age dependent susceptibility. Age dependent incubation period has recently been revived by Cooper et al., because scenarios including longer incubation periods in old cohorts provided a better fit of the data[10]. However, the increase of the mean age of cases with time which should occur with age dependent incubation periods [2], is not supported by the data. The correlation between age at onset and calendar time remains indeed extremely small (Spearman correlation coefficient r = -0.025, *n *= 129).

The potential role of differential dietary exposure was proposed early in the epidemic [12], because substantial differences in exposure existed in adolescents and adults. A disequilibrium in sex ratio towards males was for example predicted on this basis [13], but is not statistically established today (*χ*^2 ^test, P = 0.49) although the proportion of males is 55%. Our analysis shows that, while the estimated age risk function for infection with vCJD exhibits a peak in the young that was also found in dietary exposure to potentially BSE infected products, differential dietary exposure alone does not account for the observed pattern in the age of the vCJD cases. Indeed, the relative exposure does not decrease rapidly enough with age to reduce the risk in older adults. Therefore, an additional effect of age is required to fully account for the age of the vCJD cases. Once exposure is taken into account, this effect appears to be peaking in children less than 10 years old, and decreasing afterwards.

Contamination through BSE infected food is today the most plausible explanation for the occurrence of vCJD; this is the rationale for correcting the risk of infection by the level of dietary exposure. However, this explanation remains today hypothetical, because even if epidemiology and biochemistry favour a link between BSE and vCJD, this is not regarded as ultimately conclusive [14]. In this work, we examined the further possibility that, provided dietary exposure was the culprit, differences in age-related exposure could explain the age distribution of cases. This assumes that the risk of vCJD infection may have been larger in those consuming more bovine products; however population based models linking bovine products consumption to vCJD incidence were not conclusive in this respect [15].

## Conclusions

The reasons for an increased susceptibility in teenagers compared to young children and adults are today speculative. In many infectious diseases, a decrease in susceptibility with age is mediated through the immunological responses to repeated exposure since birth. However, for vCJD, most cohorts in the population have had the same duration of exposure to BSE infected material. Due to the age range considered, biological processes involved in the maturation of the immune system; in response to hormonal changes may be incriminated. For example, changes in the permeability of the intestinal barrier occurs with age with decreasing Peyer's patches [16]. Further experimental research is required to provide explanations for what remains a very unusual characteristic in an infectious disease.

## List of abbreviations

vCJD: variant Creutzfeldt-Jacob Disease

BSE : Bovine Spongiform Encephalopathy

## Competing interests

None declared.

## Authors' contributions

PYB designed the study, analysed the results and drafted the manuscript. JYC participated in the design and writing. AJV participated in the design and writing. All authors read and approved the final manuscript.

## Pre-publication history

The pre-publication history for this paper can be accessed here:
